# Efficiency and safety of Buzhong Tiaogan granule for treating the colorectal cancer patients with liver metastasis: study protocol for a multicenter randomized controlled trial

**DOI:** 10.3389/fmed.2024.1465280

**Published:** 2024-11-15

**Authors:** Shu-Lan Hao, Yan-Chen Zhou, Xiao-Li Li, Qi-Ming Zhong, Li-Kun Liu, Yu Gao, Xi-Xing Wang, Wen-Hui Yang, Li-Fang Yang

**Affiliations:** ^1^Department of Oncology, Shanxi Province Academy of Traditional Chinese Medicine & Shanxi Traditional Chinese Medical Hospital, Taiyuan, China; ^2^Department of Gastroenterology, Shanxi Cancer Hospital Hospital, Taiyuan, China; ^3^Department of Gastroenterology, Affiliated Hospital of Shanxi University of Chinese Medicine, Taiyuan, China

**Keywords:** colorectal cancer, liver metastasis, Buzhong Tiaogan granule, randomized controlled trial, traditional Chinese medicine, clinical protocols

## Abstract

**Objective:**

To evaluate the clinical efficacy and safety of Buzhong Tiaogan granule (BTG) in treatment of colorectal cancer patients with liver metastasis and provide high-level evidence for clinical practice.

**Methods and analysis:**

This is a prospective, randomized, controlled, multi-center controlled trial. A total of 210 patients diagnosed with spleen deficiency and liver depression, as well as colorectal cancer liver metastasis (CRLM) of the stasis and toxin interception type, will be enrolled in the study. The participants will be randomly allocated into two groups: a treatment group that will receive BTG plus conventional first-line anti-tumor therapy and a control group that will receive conventional first-line anti-tumor therapy alone. The primary outcomes are progression-free survival (PFS) and quality of life scores. The secondary outcomes are as follows: (a) overall survival (OS); (b) objective response rate (ORR); (c) liver-specific progression-free survival; (d) disease control rate (DCR); (e) traditional Chinese medicine (TCM) syndrome score; (f) Piper Fatigue Scale, and (g) Karnofsky Performance Status (KPS) score. Safety evaluations will be conducted throughout the study period.

**Discussion:**

The results of this trial will provide scientific and objective data necessary for the evaluation of the efficacy and safety of BTG for colorectal cancer patients with liver metastasis.

**Clinical trial registration:**

The trial was registered at ClinicalTrials.gov (ChiCTR2400084861) on May 27, 2024.

## Introduction

Colorectal cancer (CRC) represents the third most prevalent form of cancer globally and the fourth leading cause of cancer-related mortality, accounting for 9.6% of global cancer-related deaths (1, 2). The five-year overall survival rate for patients with metastatic colorectal cancer is approximately 13% (3). The liver is the primary site of metastases in colorectal cancer, with liver metastases representing the most common cause of death in these patients. It is estimated that 30–40% of all patients diagnosed with CRC will develop colorectal cancer liver metastasis (CRLM) over the course of their disease (4). Liver resection represents the gold standard of treatment for patients with CRLM, offering the most favorable prognosis (5). However, due to significant complications, including liver failure, blood loss, and bile leaks, the five-year survival rates following surgery are only 30 to 50%. Furthermore, more than 70% of patients will experience a recurrence following resection for CRLM (6). Consequently, the search for an efficacious treatment schedule has become a significant research focus for scholars at both national and international levels.

The treatment of metastatic colorectal cancer (mCRC) represents a significant clinical challenge (7). It has been estimated that between 20 and 25% of patients with stage IV CRC have synchronous distant metastasis, with CRLM accounts for approximately 45% of mCRC cases (8). Moreover, it is estimated that 70–75% of patients with CRLM are unable to tolerate liver resection surgery, or their liver metastasis remains unresectable following systemic therapy (9). The most commonly employed chemotherapeutic agents for the treatment of CRLM include fluorouracil, irinotecan, oxaliplatin, and raltitrexed (10). The aforementioned chemotherapy drugs are frequently used in combination, with the most commonly employed combination chemotherapy regimens including FOLFOX, FOLFIRI, CAPEOX, and FOLFOXIRI, among others (11). The application of molecularly targeted drugs has resulted in notable benefits for patients with CRC. At present, the most frequently used molecularly targeted drugs in the clinical treatment of CRC are anti-angiogenesis agents, represented by bevacizumab, and anti-epidermal growth factor drugs, exemplified by cetuximab (12). In the clinical setting, the use of molecularly targeted drugs is typically recommended in combination with chemotherapeutic drugs. Patients undergoing chemotherapy frequently experience significant adverse events, with 10–27% encountering grade 3 or grade 4 complications, including neutropenia, skin toxicity, liver toxicity, disease progression during therapy, secondary splenomegaly, and the selection of resistant tumor clones (13). A prolonged duration of preoperative chemotherapy is associated with an increased risk of liver toxicity and postoperative complications. It is recommended that chemotherapy be restricted to four to six cycles for patients scheduled for subsequent liver resection (14). It is evident that there is a significant unmet medical need for the development of more efficacious treatment strategies and medical interventions, particularly those with high efficacy and minimal side effects.

Traditional Chinese Medicine (TCM) represents a significant source of natural medicines and herbal products and is therefore an indispensable component in the development of anti-CRC agents (15). Recent studies have demonstrated that TCM can be an effective supplementary method for reducing the incidence of CRC (16). The active components of Chinese herbs have been demonstrated to disrupt the living environment of cancer cells, promote apoptosis, and enhance individual immunity. The collective regulatory impact of these herbs has the potential to markedly enhance the quality of life and survival rate of CRC patients, thereby achieving an anticancer effect (17). Moreover, the combination of TCM with other chemotherapy agents has the potential to mitigate the adverse effects of chemotherapy, thereby enhancing the quality of life of patients. For example, the Shaoyao decoction has been demonstrated to significantly reduce the prevalence of colonic neoplasms (18). Furthermore, it has been reported that the Da Huang Zhe Chong pill inhibits the progression of CRC to the liver by modifying the exosomal CCL2-primed premetastatic niche (19). A recent study demonstrated that erinacines from *Hericium erinaceus* have been shown to reduce the inflammatory burden on the intestinal mucosa of patients with inflammatory bowel disease (IBD), a critical factor in the development of inflammation-induced colon cancer (20). Despite the aforementioned benefits, the potential of TCM in the treatment of CRLM remains under-researched. It is therefore necessary to identify more effective and diverse methods of TCM treatment for CRLM.

TCM syndromes form the core foundation for understanding diseases, clinical diagnosis, and evaluating treatment efficacy in TCM practice (21). Under the theoretical framework of syndrome differentiation-based treatment, TCM demonstrates distinct characteristics and advantages, particularly in the management of complex and refractory diseases such as malignant tumors (22). Buzhong Tiaogan granule (BTG) is a Chinese herbal formula comprising 14 distinct herbs, including *Astragalus* 60 g, *Codonopsis pilosula* 15 g, *fried Atractylodes* 15 g, *Cimicifugae rhizoma* 6 g, *Bupleuri radix* 10 g, *Angelicae radix* 10 g, *Citri reticulatae pericarpium* 10 g, *Paeoniae alba radix* 15 g, *Fritillariae thunbergii bulbus* 30 g, *Salvia chinensis* 60 g, *Centipede* 6 g, *Pleione yunnanensis* 30 g, *Prunella vulgaris* 30 g, and *Glycyrrhiza glabra* 6 g. In China, TCM is frequently employed as an adjuvant chemotherapy to mitigate adverse effects and enhance the completion rate of chemotherapy. BTG is a well-established formula that has been employed by our team for over two decades in the treatment of CRLM (23). A study by Li et al. (24) indicates that BTG can strengthen vital qi and eliminates pathogens, which could potentially improve fever, bleeding, myelosuppression and the completion rate of chemotherapy. However, there is a paucity of evidence regarding the efficacy of this treatment in delaying the development of colorectal liver metastasis, particularly in patients presenting with liver depression and spleen deficiency. So, the purpose of this study is to assess the efficacy and safety of BTG in conjunction with chemotherapy in improving progression-free survival (PFS) and quality of life in patients with CRLM.

## Materials and methods

### Study design

**Figure 1:**
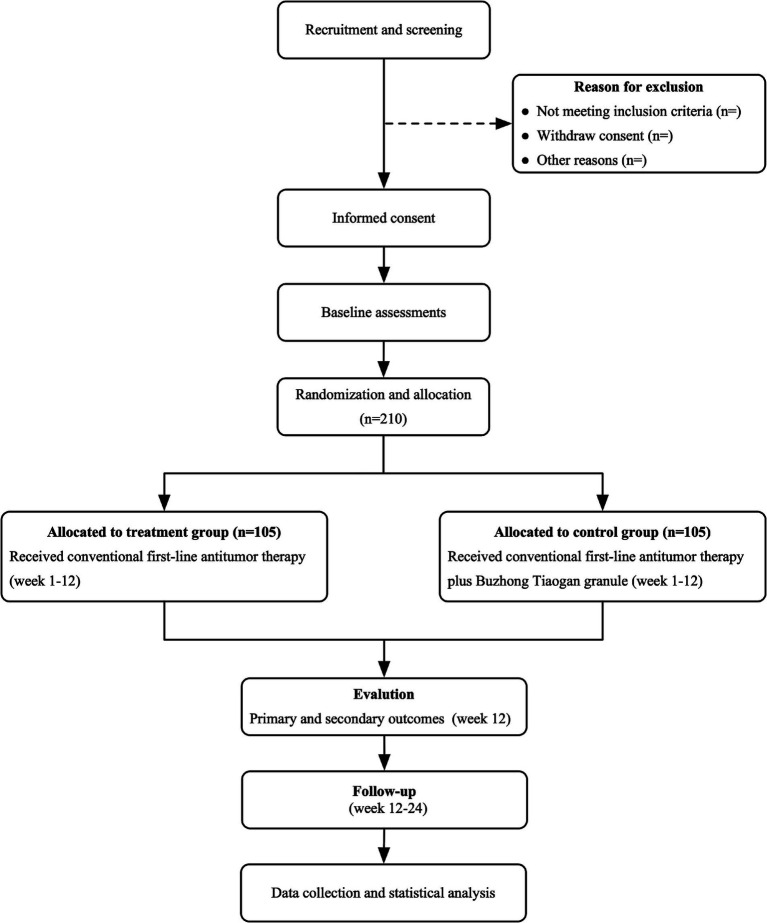
Trial flow chart.

This study is a prospective, randomized, controlled, multicenter clinical trial evaluating the efficacy of BTG in combination with chemotherapy for the treatment of CRLM characterized by spleen deficiency and liver depression, stasis, and toxin interpunction type. Participants will be divided into two distinct groups: the treatment group, which will receive conventional first-line antitumor therapy in combination with BTG, and the control group, which will receive conventional first-line antitumor therapy alone. Patients will be treated until disease progression is observed, at which point they will be followed up until death. The trial design is presented in Figure 1 and the schedule in Table 1, respectively.

**Table 1:** Schedule of enrollment, assessments, and interventions.

Item	Study period			
	Enrollment	Allocation	Post-allocation	Close-out
Time point (week)	−4–0	0	1–12	12–24
Enrolment				
Eligibility screen				
Informed consent	×			
Inclusion/exclusion	×			
Randomization		×		
Allocation		×		
Intervention				
BTG plus standard treatment			×	
Standard treatment			×	
Assessment				
PFS			×	×
Quality of life scores			×	×
Overall survival			×	×
Objective response rate			×	×
Liver-specific PFS			×	×
Disease control rate			×	×
TCM syndrome score			×	×
Piper Fatigue Scale			×	×
KPS			×	×
Adverse events			×	×
Compliance evaluation				×
Acceptability evaluation				×

BTG, Buzhong Tiaogan granule; TCM, Traditional Chinese Medicine; KPS, Karnofsky performance score; PFS, Progression-free survival.

### Ethics approval and consent to participate

This randomized controlled trial has been approved by the Human Research Ethics Committee of Shanxi hospital of Traditional Chinese medicine (approval No. 2024KY-07020) and registered in the Chinese Clinical Trial Registry (No. ChiCTR2400084861). Prior to participation in any aspect of the study, all participants will be required to provide written informed consent. Moreover, participants may withdraw from the study at any point. Following the assessment of relevant entry criteria within a week preceding the commencement of the intervention, enrolment will be confirmed. At this point, baseline characteristics, including sex, age, body mass index (BMI), pathological type, Eastern Cooperative Oncology Group (ECOG) performance status score, and disease history, will be recorded. A series of tests will be conducted on each participant, including full blood count, urine analysis, tumor markers analysis (e.g., CEA and CA19–9), liver and renal function tests. These tests will be performed at the baseline, treatment weeks 3, 6, 9, and 12.

### Inclusion criteria

Participants will be included if they meet the diagnosis criteria of the 2023 edition of the Colorectal Cancer Diagnosis and Treatment Guidelines issued by the Chinese Society of Clinical Oncology (CSCO) (25) and the 2008 edition of the Guidelines for the Diagnosis and Treatment of Cancer in TCM issued by the Chinese Association of Traditional Chinese Medicine (26).

Participants are eligible to participate in this study after meeting all of the following criteria: (a) aged 18–80 years, male or female, with an expected survival of ≥3 months; (b) diagnosed with colorectal adenocarcinoma, deemed unresectable by multidisciplinary assessment, with liver-only or liver-based metastasis; (c) evaluable liver metastases (maximum diameter ≥ 10 mm) unsuitable for surgical resection or local ablation; (d) no prior advanced systemic or local therapy for liver metastases; (e) diagnosed with spleen deficiency and liver depression, stasis, and toxin interpunction per TCM criteria; (f) Karnofsky Performance Status (KPS) score ≥ 60; (g) voluntarily participate in the study, and patients or their immediate relatives are able and willing to sign the informed consent.

### Exclusion criteria

Participants who meet any of the following criteria will be excluded from this study: (a) non-primary colorectal cancer with an unclear diagnosis; (b) with polygenic carcinoma or other malignant neoplasms; (c) extensive peritoneal metastases, MSI-H/dMMR, or other targeted therapies; (d) presenting with severe hepatic or renal insufficiency, intestinal obstruction, perforation, jaundice, or bleeding; (e) unwilling or unable to accept TCM treatment; (f) allergy to one or more of the Chinese herbs included in this protocol; (g) pregnancy or lactation in women; (h) poor compliance or inability to commit to completing the protocol; (i) participation in other clinical trials within the previous 3 months.

### Criteria for withdrawal

Participants will freely withdraw (discontinue participation) from the trial at any time and for any reason. Researchers should meet all research expectations, including regulatory requirements in the current trial and the Guidelines for Good Clinical Practice. Researchers should prevent patients from the situation such as (but not limited to): occurrence of an unexpected serious concomitant disease.

### Sample size

A review of previous literature indicates that the median progression-free survival (PFS) of the control group is estimated to be 6.1 months. With a significance level of *α* = 0.05 (two-sided), a power of 0.9.

Sample size is calculated according to the following formula:

$n={\left(Z_{1-\alpha }+Z_{1-\beta }\right)}^{2}\times \left[\pi_{1}\times \left(1-\pi_{1}\right)+\pi_{2}\times \left(1-\pi_{2}\right)\right]/{\left(\pi_{1}-\pi_{2}\right)}^{2}\text{.}$

Considering a dropout rate controlled within 10%, the sample size calculation indicates that 105 cases per group, totaling 210 cases, are required.

### Randomization and blinding

Participants will be randomly assigned to treatment and control groups in a 1:1 ratio using SAS9.3 software to generate the random sequence in blocks of 6 with no stratification. An independent researcher, not involved in treatment or data analysis, will generate the sequence. Randomization assignments were concealed in opaque, sealed envelopes, which were distributed sequentially to participants after informed consent was obtained and baseline measurements were completed. Randomization will be managed through a central system, with clinical investigators accessing it by telephone or online to obtain randomization numbers. This study employed a single-blind design. The investigator responsible for patient assignment was not involved in patient recruitment, assessment, treatment, or data analysis. Study evaluators remained blinded to patient allocation throughout the study.

### Interventions

All patients will receive a standard chemotherapy regimen following the initial treatment. The chemotherapy regimen is XELOX (capecitabine plus oxaliplatin), administered over four cycles spanning 12 weeks, with each cycle lasting 3 weeks. The XELOX regimen is as follows: oxaliplatin will be administered intravenously at a dose of 150 mg/m^2^ on Day 1 of each 3-week cycle, and capecitabine will be administered orally at a dose of 1,000 mg/m^2^, twice daily, from Day 1 to Day 14 of each 3-week cycle. The control group will receive only the standard XELOX chemotherapy regimen. In contrast, the treatment group will receive the standard XELOX chemotherapy along with BTG. BTG will be administered orally at a dose of 10 g, twice daily, from Day 1 to Day 14 of each 3-week cycle. Chemotherapy will be administered in the outpatient clinic, and patients will undergo follow-up assessments at three-week intervals. All patients will have weekly clinical evaluations and routine blood tests throughout the chemotherapy course. Supportive therapies, including myocardial protection, liver protection, acid inhibition, and antiemetic treatments, will be provided as needed to manage potential adverse effects.

### Primary outcomes

The primary outcomes will be the PFS, with quality of life as a co-primary outcome. PFS will be calculated as the time from randomization to the first recurrence, metastasis, progression, or death from any cause, in accordance with the Response Evaluation Criteria in Solid Tumors (RECIST), version 1.1. Quality of life will be assessed using the European Organization for Research and Treatment of Cancer Quality of Life Questionnaire C30 (EORTC QLQ-C30).

### Secondary outcomes

Secondary outcomes will include the overall survival (OS), objective response rate (ORR), disease control rate (DCR), liver-specific progression-free survival, efficacy of TCM syndrome, piper fatigue scale, and Karnofsky performance status (KPS) score. OS is analyzed by calculating the time interval from the date of inclusion to the date of death from any cause or the date of the last follow-up for surviving patients, and then estimated using the Kaplan–Meier method. Objective tumor responses were evaluated based on complete response (CR), partial response (PR), stable disease (SD), and progressive disease (PD). ORR is defined as the proportion of patients achieving CR and PR. DCR is calculated as the combined proportion of patients with CR, PR, and SD. Liver-specific progression-free survival is defined as the time from randomization to radiological liver progression. The TCM syndrome therapeutic effect referred to the judgment standard of TCM syndromes of cancer-related fatigue according to the Principles of Clinical Research Guidelines for New Chinese Drugs (27). The therapeutic effect index = (pretreatment total score − posttreatment total score)/pre-treatment total score × 100%. The definitions of clinical efficacy included healed (efficacy index ≥90%), markedly effective (efficacy index <90% and ≥ 70%), effective (efficacy index <70% and ≥ 30%) and ineffective (efficacy index <30%). The therapeutic effect was evaluated by the percentage of TCM syndrome scoring of the patients. Total efficiency = (healed number + markedly effective number + effective number)/total number × 100%.

### Incidence of adverse events

All adverse events (AEs) will be recorded and assessed. AEs occurring during the course of treatment will be classified in accordance with the US National Cancer Institute Common Terminology Criteria for Adverse Events (version 4.0) or World Health Organization (WHO) grading. Once grade 3–4 AEs occur, a comprehensive account will be compiled, including the time of occurrence, clinical manifestations, duration, physical examination results, laboratory findings, interventions and outcome. Researchers will discuss the causal relationship with the testing treatment and decide whether participation in the trial should continue.

### Follow-up

In all centers, we intend to conduct long-term follow-up (12–24 weeks post-treatment) beyond the duration of this project to monitor metastasis development in accordance with current standards. Data on disease-free survival, cancer-specific survival, and overall survival will be recorded.

### Quality control and data management

All study data will be entered into electronic case report forms within the study’s electronic data capture system to facilitate data integration and management. The system includes a complete audit trail, utilizing anonymous subject identification numbers. The principal study site will establish a unified data processing agreement with collaborating centers and ensure strict adherence to its implementation. Any amendments to the protocol must be submitted to and approved by the Ethics Committee of Shanxi hospital of traditional Chinese medicine. At the end of the study, the database will be locked and analyzed in accordance with the agreement between the principal investigator and statistician, who will be granted access to the final trial dataset.

### Statistical analysis

The EpiData 3.0 software will be employed for the management of the database, while the SAS 9.2 software will be utilized for the statistical analysis. The study is conducted in accordance with intention-to-treat (ITT) analysis principles. Continuous data that conforms to a normal distribution will be represented by the mean ± standard deviation. Hypothesis testing will be conducted using an independent sample *t*-test. In the event that the data does not conform to a normal distribution, it will be represented by the median (interquartile range) and analyzed using the Kruskal-Wallis test. In the context of multicenter analysis, the CMH chi-squared test and covariance analysis will be employed. PFS will be analyzed using Cox regression, while TCM symptom scores, quality of life scores, and Piper Fatigue Scale data will be analyzed using covariance analysis. The Wilcoxon rank-sum test will be employed to assess the efficacy of TCM symptoms. The differences between the treatment and control groups will be quantified using the log-rank method. Missing data were addressed using a simple imputation method. No interim analyses were planned or conducted. Subgroups analysis will be carried out by the left or right colon, stages of CRLM, sex, and age. A *p*-value of less than 0.05 will indicate statistical significance.

## Discussion

Liver metastasis represents a significant contributor to mortality in patients diagnosed with CRC (28). Radical surgery has been demonstrated to result in a superior prognosis and offers patients a high quality of life in cases where the tumor is resectable. Nevertheless, only approximately 10–20% of patients with CRLM are suitable for surgical intervention (29). It has been estimated that between 30 and 50% of patients with CRC experience recurrent liver metastasis following radical resection, with a mortality rate exceeding 50% (30). Systemic chemotherapy represents the standard of care for inoperable patients with inoperable liver metastases from colorectal cancer. The combination of oxaliplatin or irinotecan with 5-fluorouracil and leucovorin has been shown to improve the response rate from 20 to 50% (31). The administration of targeted drugs, such as bevacizumab or cetuximab, has been demonstrated to enhance the response rate by up to 70% (32). Nevertheless, the current therapeutic approaches for patients with CRLM, including surgery, radiotherapy, and chemotherapy, remain suboptimal due to the rapid progression of metastasis and the development of drug resistance.

According to TCM theory, the spleen is deemed to be the organ responsible for providing the material basis for the acquired constitution, as well as being the source of Qi and blood (33). CRC is situated within the bowel and is closely associated with the function of the spleen. Patients with colorectal cancer and liver metastasis frequently present with symptoms of spleen and Qi blood deficiency, including fatigue, poor appetite, and abdominal distension (34). Patients with colorectal cancer and liver metastasis present with both deficiency and excess syndromes, necessitating a treatment approach that balances replenishing deficiency and expelling pathogenic factors. The theory of TCM maintains that while chemotherapy is an effective method of eradicating pathogenic agents, it can also cause damage to the viscera, bone marrow, blood, yin, and yang, thereby impairing the functions of the spleen, stomach, liver, and kidney (35). The therapeutic approach should prioritize the supplementation of the middle Qi, the regulation of the liver, the tonification of Qi, and the nourishment of yin. This should be accompanied by the elimination of toxins and the dissolution of masses, with the objective of alleviating symptoms and preventing disease progression. BTG is a Chinese medicinal formulation designed by the national medical master, professor Xixing Wang. It is based on Li Dongyuan’s Buzhong Yiqi decoction, as described in the Treatise on Internal and External Damage and Confusion. *Astragalus* is employed to enhance vital Qi, with *Codonopsis pilosula* and *angelica radix* serving to supplement both Qi and blood, thereby providing continuous energization of the middle burner. This is facilitated by the *Cimicifuga rhizoma* and *Bupleurum radix*, which elevate clear yang, thus relieving fatigue and improving physical condition. *Codonopsis pilosula*, fried *Atractylodes*, and *Citri reticulatae pericarpium* are employed to fortify the spleen and stomach, enhance digestive processes, and mitigate the symptoms of poor appetite and nausea. The combination of *Astragalus* and *Bupleurum radix exerts* a tonifying effect on the spleen and a soothing effect on the liver, thereby harmonizing the movement of Qi. *Glycyrrhiza glabra* and *Paeoniae alba radix* possessed relaxing properties on the liver, alleviating spasms and providing relief from abdominal pain and bowel sounds. BTG has been demonstrated to be an effective treatment for patients with liver-spleen deficiency and phlegm stasis toxin accumulation type CRLM, providing relief from discomfort and improving physical condition (23).

It is important to acknowledge two significant limitations. Firstly, the limited availability of funding restricts the scope of large-scale sample studies, which may result in a reduction in the level of evidence in this trial. Nevertheless, we will endeavor to enhance patient compliance in order to optimize the quality of the research. Secondly, as this is a multicenter clinical trial, the main research center, which has extensive clinical experience, may encounter fewer practical issues. Nevertheless, the implementation of this therapy in other participating centers may present novel challenges.

The results of this RCT will contribute to clinical practice by assessing the efficacy of BTG in delaying CRLM. Additionally, the study will provide further support for the broader implementation of BTG in clinical settings for patients with CRLM in China.

### Trial status

Currently, participant recruitment is ongoing.
